# Supplementary material for: Throughput screening of Bacillus subtilis strains that abundantly secrete surfactin in vitro identifies effective probiotic candidates
Source: PLoS One. 2022 Nov 23;17(11):e0277412. doi: 10.1371/journal.pone.0277412 (PMC9683610; doi:10.1371/journal.pone.0277412)

Supplemental Figure 1. We fractionated the Proxenrem surfactin into four major surfactin isoforms A, B, C, and D using the C18 column by a preparative HPLC system.(Agilent Technologies, Santa Clara, CA, USA).


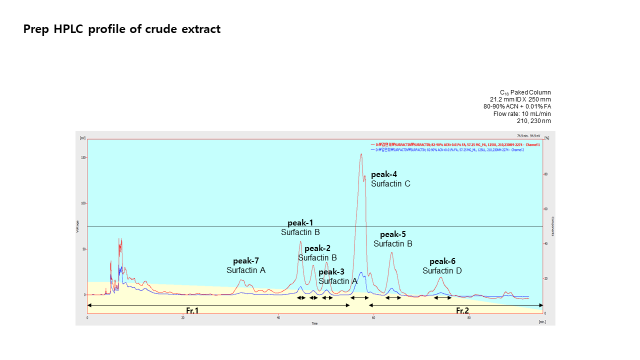


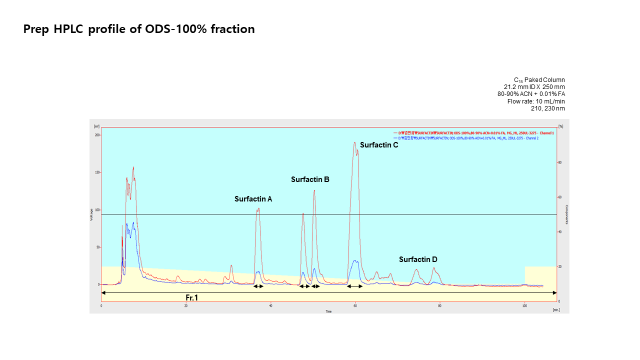


Supplemental Figure 2. Maximum likelihood phylogeny constructed with 1,162 orthologous genes shared among all taxa.


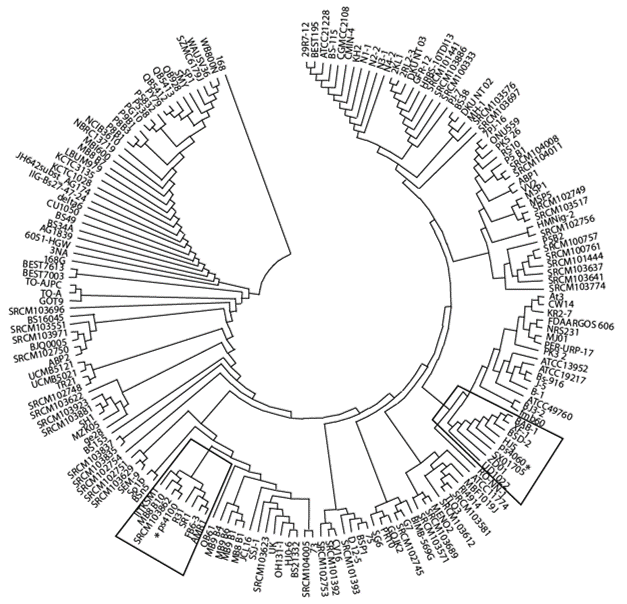

Supplement: S1 File — (DOCX) [file pone.0277412.s001.docx]
