# Supplementary figures and images for: Throughput screening of Bacillus subtilis strains that abundantly secrete surfactin in vitro identifies effective probiotic candidates
Source: PLoS One. 2022 Nov 23;17(11):e0277412. doi: 10.1371/journal.pone.0277412 (PMC9683610; doi:10.1371/journal.pone.0277412)

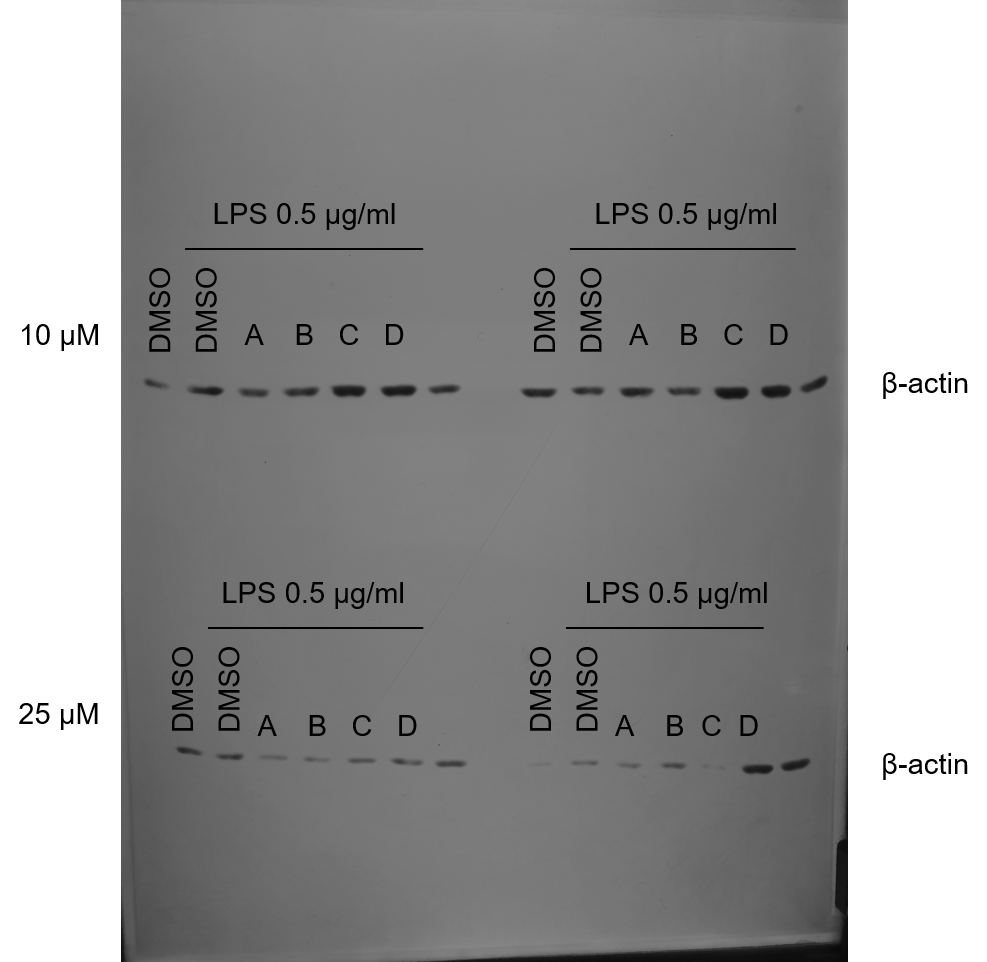

Supplement: S2 File — (PNG) [file pone.0277412.s002.png]

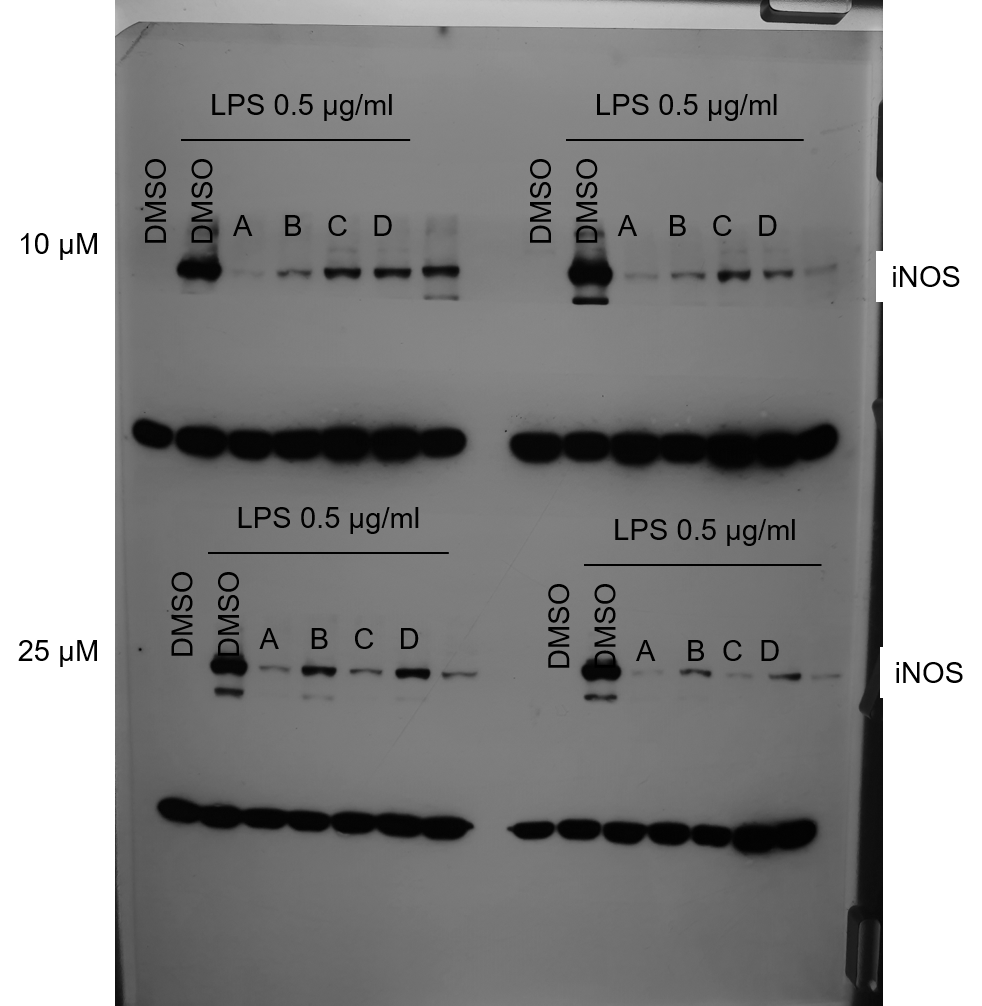

Supplement: S3 File — (PNG) [file pone.0277412.s003.png]
